# Liver Stiffness-Based Risk Prediction Model for Hepatocellular Carcinoma in Patients with Nonalcoholic Fatty Liver Disease

**DOI:** 10.3390/cancers13184567

**Published:** 2021-09-11

**Authors:** Jae Seung Lee, Dong Hyun Sinn, Soo Young Park, Hye Jung Shin, Hye Won Lee, Beom Kyung Kim, Jun Yong Park, Do Young Kim, Sang Hoon Ahn, Joo Hyun Oh, Jung Il Lee, Seung Up Kim

**Affiliations:** 1 Department of Internal Medicine, Yonsei University College of Medicine, Seoul 03722, Korea; sikarue@yuhs.ac (J.S.L.); lorry-lee@yuhs.ac (H.W.L.); beomkkim@yuhs.ac (B.K.K.); drpjy@yuhs.ac (J.Y.P.); dyk1025@yuhs.ac (D.Y.K.); ahnsh@yuhs.ac (S.H.A.); 2 Institute of Gastroenterology, Yonsei University College of Medicine, Seoul 03722, Korea; 3 Yonsei Liver Center, Severance Hospital, Seoul 03722, Korea; 4 Department of Medicine, Samsung Medical Center, Seoul 06351, Korea; sinndhn@hanmail.net; 5 Department of Internal Medicine, School of Medicine, Kyungpook National University Hospital, Daegu 41944, Korea; psyoung0419@gmail.com; 6 Biostatistics Collaboration Unit, Department of Biomedical Systems Informatics, Yonsei University College of Medicine, Seoul 03722, Korea; HJSHIN105@yuhs.ac; 7 Department of Medicine, Nowon Eulji Medical Center, Seoul 01830, Korea; ojh8856@gmail.com; 8 Department of Internal Medicine, Gangnam Severance Hospital, Seoul 06273, Korea

**Keywords:** hepatocellular carcinoma, non-alcoholic fatty liver disease, liver cirrhosis, liver fibrosis, transient elastography, liver stiffness, risk prediction

## Abstract

**Simple Summary:**

A new liver stiffness (LS) based risk prediction model for the development of hepatocellular carcinoma (HCC) in patients with non-alcoholic fatty liver disease (NAFLD), which consists of old age, low platelet count, aspartate aminotransferase level, and high LS measured by transient elastography, showed acceptable performance in the internal and external validation in Asian patients.

**Abstract:**

Non-alcoholic fatty liver disease (NAFLD) is associated with an increased hepatocellular carcinoma (HCC) risk. We established and validated a liver stiffness (LS)-based risk prediction model for HCC development in patients with NAFLD. A total of 2666 and 467 patients with NAFLD were recruited in the training and validation cohorts, respectively. NAFLD was defined as controlled attenuated parameter ≥238 dB/m by transient elastography. Over a median of 64.6 months, HCC developed in 22 (0.8%) subjects in the training cohort. Subjects who developed HCC were older and had higher prevalence of diabetes and cirrhosis, lower platelet count, and higher AST levels compared to those who did not develop HCC (all *p* < 0.05). In multivariate analysis, age ≥60 years (hazard ratio (HR) = 9.1), platelet count <150 × 10^3^/μL (HR = 3.7), and LS ≥9.3 kPa (HR = 13.8) were independent predictors (all *p* < 0.05) that were used to develop a risk prediction model for HCC development, together with AST ≥34 IU/L. AUCs for predicting HCC development at 2, 3, and 5 years were 0.948, 0.947, and 0.939, respectively. This model was validated in the validation cohort (AUC 0.777, 0.781, and 0.784 at 2, 3, and 5 years, respectively). The new risk prediction model for NAFLD-related HCC development showed acceptable performance in the training and validation cohorts.

## 1. Introduction

Currently, non-alcoholic fatty liver disease (NAFLD) is known to affect about one quarter of the global population [1]. The prevalence of NAFLD in the Republic of Korea (ROK) is also rapidly increasing, and is estimated to account for approximately 30% of the general population in the country [2,3]. Approximately 20% of the patients with NAFLD develop nonalcoholic steatohepatitis (NASH), a chronic inflammatory condition that is associated with altered lipid metabolism, and has an increased risk of cirrhosis and hepatocellular carcinoma (HCC) [4,5].

HCC is one of the leading causes of cancer death worldwide [6]. In the ROK, the number of annual deaths and crude death rate have been reported to increase since 2013 due to liver cancer [7], which is still the second largest cause of cancer mortality and the most economically burdensome cancer [8]. Indeed, the prevalence of NAFLD-related HCC appears to have increased over time [4,9,10].

Among the possible risk factors such as old age, diabetes mellitus (DM), alcohol consumption and elevated aspartate aminotransferase (AST), the presence of NASH and cirrhosis are known to be the most powerful risk factors for HCC development in NAFLD [11,12,13]. Of these, NASH is based on histological diagnosis, and cirrhosis is based on ultrasonography (US), which might miss advanced liver fibrosis or early compensated liver cirrhosis [14]. Recently, liver stiffness (LS) measurement using transient elastography (TE) has been proven to be a useful tool to assess the risk of NASH and degree of fibrotic burden in the liver, which are significantly associated with the risk of HCC development [15,16,17,18]. However, no risk prediction model for NAFLD-related HCC, especially one that is based on LS assessment, has been available.

Therefore, in this study, we aimed to identify independent predictors of HCC development and also establish and validate a risk prediction model for HCC development in patients with NAFLD.

## 2. Materials and Methods

### 2.1. Patient Eligibility

Between March 2012 and June 2020, subjects who were diagnosed with controlled attenuated parameter (CAP)-based NAFLD were recruited through a retrospective review using the consecutively registered databases at three high-volume medical centers (Severance Hospital, Samsung Medical Center, and Gangnam Severance Hospital) as a training cohort. Data from Kyungpook National University Hospital were included as a validation cohort.

**Figure 1:**
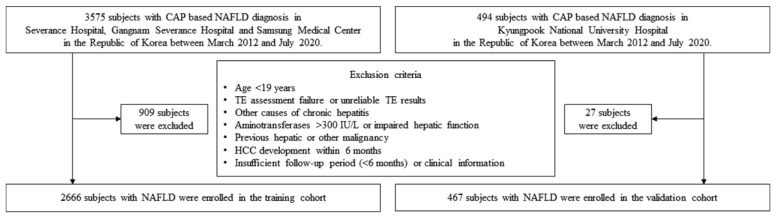
Flow chart of study subject selection. CAP, controlled attenuated parameter; NAFLD, non-alcoholic fatty liver disease; TE, transient elastography; IQR, interquartile range; HCC, hepatocellular carcinoma.

The exclusion criteria of both cohorts were as follows: (1) age < 19 years; (2) TE assessment failure using M probe; (3) unreliable TE results; (4) other causes of chronic hepatitis such as hepatitis B, hepatitis C, autoimmune hepatitis, primary biliary cholangitis and overlap syndrome; (5) high aminotransferase levels (>300 IU/L); (6) impaired hepatic function such as high total bilirubin (>3.0 mg/dL) and low albumin (<2.5 g/dL); (7) HCC development within 6 months after enrollment; (8) liver transplantation within 6 months after enrollment; (9) insufficient follow-up period of less than 6 months; and (10) insufficient clinical information (Figure 1).

### 2.2. Definition

The presence of fatty liver was defined as an elevated CAP ≥ 238 dB/m by TE (EchoSens, Paris, France) [19,20,21]. The first date of the diagnosis of NAFLD based on TE was defined as the index date. The presence of cirrhosis was defined to as findings suggestive of cirrhosis, including a blunted, nodular liver edge accompanied by splenomegaly (>12 cm) in the imaging studies such as US, computed tomography (CT) or magnetic resonance imaging (MRI) that were performed close to the index date.

### 2.3. TE Assessment

At each hospital, TE was performed by experienced operators who had conducted at least 500 examinations. Patients were examined after overnight fasting using M probes, considering the patients’ body mass indices. LS (kPa) and CAP (dB/m) measurements were recorded until 10 valid measurements were obtained for each patient. The median value was considered representative of the elastic modulus of the liver. Only procedures with at least 10 valid measurements, a success rate of at least 60%, and an interquartile range (IQR) to median value ratio of 30% were considered reliable [22,23].

### 2.4. HCC

The outcome of this study was the histological or clinical diagnosis of HCC according to the timely guidelines proposed by the Korea Liver Cancer Study group [24]. A positive finding for a typical HCC on dynamic CT or MRI was indicated by an increased arterial enhancement followed by a decreased enhancement compared with the liver (washout) in the portal or equilibrium phase.

### 2.5. Statistical Analyses

Continuous variables such as laboratory test results are expressed as the medians (interquartile ranges (IQRs)) and were compared using Student’s t-test or Mann–Whitney U test depending on their distribution. Categorical variables are expressed as numbers (and percentages) and were evaluated using chi-square test or Fisher’s exact probability test. Patients were censored when they ended follow-up, died without developing liver cancer, or developed extrahepatic carcinoma. Both univariate and multivariate Cox regressions were employed to analyze the association between HCC development and risk factors, and to calculate their hazard ratios (HRs) with 95% confidence intervals (CI). Continuous variables were categorized by the Youden index or cutoffs that are clinically in use. The presence of cirrhosis by imaging studies was not considered in the Cox regression analysis because of the possibility of multicollinearity with LS. In the multivariate Cox regression model, we considered some known risk factors in the previous reports such as old age, DM and elevated AST [11,12,13,25]. Only few factors were considered in the multivariate Cox regression model due to the small number of events [25]. The predictive ability of the model was assessed by the integrated area under the curve (iAUC) and time-dependent area under the curve (AUC) at 2, 3, and 5 years after the index date [26]. The model performance was presented graphically by calibration plots which compared the model prediction probability with the actual probability. The risk prediction model was rigorously assessed using an internal validation using a bootstrapping method and an external validation using data from the validation cohort.

Statistical analyses were performed using SAS version 9.4 (SAS Institute Inc., Cary, NC, USA) and R package (v. 4.0.4, http://www.r-project.org/, accessed on 26 July 2021) software. Two-sided *p* values < 0.05 were considered to indicate statistical significance.

## 3. Results

### 3.1. Baseline Characteristics

The flow chart of patient selection is shown in Figure 1. A total of 3575 subjects who were diagnosed with NAFLD based on TE (CAP ≥ 238 dB/m) were considered eligible. After excluding 909 subjects who met the exclusion criteria, 2666 with NAFLD were finally included in training cohort. Similarly, 494 subjects who were diagnosed NAFLD were recruited in the validation cohort. After excluding 27 subjects who met the exclusion criteria, 467 patients were finally included in the validation cohort.

Baseline characteristics of the training cohort are presented in Table 1. At the index date, the median age of 1524 (57.2%) male subjects and 1142 (42.8%) female subjects was 52.0 (IQR 41.0–60.0) years. DM, hypertension and cirrhosis were observed in 1029 (38.6%), 1080 (40.5%) and 171 (6.4%) subjects, respectively. TE at the index date showed that the median LS and CAP were 5.9 (IQR 4.6–7.9) kPa and 303 (IQR 273–331) dB/m.

Subjects in the validation cohort (*n* = 467) showed a higher proportion of cirrhosis (9.4% (44/467) vs. 6.4% (171/2666)), higher median LS value (6.5 vs. 5.9 kPa), higher AST levels (47 vs. 33 IU/L) and higher alanine aminotransferase levels (57 vs. 41 IU/L) compared to those in the training cohort (all *p* < 0.05) (Table S1).

### 3.2. Comparison between Subjects Who Developed HCC and Those Who Did Not

During the mean follow-up period of 60.7 ± 25.7 months in the training cohort, HCC developed in 22 (0.8%) subjects (approximately 1.63 per 1000 patient years). The cumulative 2-, 3- and 5-year HCC incidence rates in the training cohort were 8 (0.3%), 13 (0.5%), and 17 (0.6%), respectively. The subjects who developed HCC (*n* = 22, 0.8%) showed significantly older age (median 70.0 vs. 52.0 years); higher prevalence of DM (59.1% vs. 38.4%), hypertension (68.2% vs. 40.3%), and cirrhosis (81.8% vs. 5.8%); higher LS value (median 21.5 vs. 5.9 kPa); lower platelet count (median 142.5 vs. 237.0 × 10^3^/μL); and higher AST levels (median 47 vs. 33 IU/L), compared to those who did not develop HCC (all *p* < 0.05) (Table 1).

In addition, during the mean follow-up period of 28.2 ± 20.8 months, nine (1.9%) subjects developed HCC in the validation cohort (approximately 8.19 per 1000 patient years).

### 3.3. Risk Factors of HCC Development in the Training Cohort

Univariate Cox regression analysis revealed that variables such as age, presence of cirrhosis, higher LS, lower platelet count, higher AST level, lower serum albumin, higher total bilirubin and higher total cholesterol were significantly associated with HCC development (all *p* < 0.05) (Table S2). Subsequent multivariate analyses based on significant variables in the univariate analysis revealed three variables, including age ≥ 60 years (HR = 9.143 (95% CI 2.055–40.684, *p* = 0.004), platelet count < 150 × 10^3^/μL (HR = 3.670 (95% CI 1.295–10.402), *p* = 0.001), and LS ≥ 9.3 kPa (HR = 13.757 (95% CI 2.826–66.955) *p* = 0.001) that were independently associated with an increased risk of HCC development (Table 2).

### 3.4. Establishment of a New Risk Prediction Model for HCC

Based on the multivariate Cox regression analysis, three predictive models (Models 1, 2 and 3) for HCC development were established, which incorporated the three independently associated factors (age, platelet count, and categorized LS), together with AST level (>34 IU/mL), which is a closely associated with HCC risk among patients with NAFLD [13] (Tables S3 and S4). The 2-, 3- and 5-year AUCs and iAUC for predicting HCC development in the training cohort were 0.943, 0.941, 0.933 and 0.939 in Model 1, respectively; 0.948, 0.947 0.939 and 0.944 in Model 2, respectively; and 0.945, 0.944, 0.940 and 0.942 in Model 3, respectively (Table S5).

**Figure 2:**
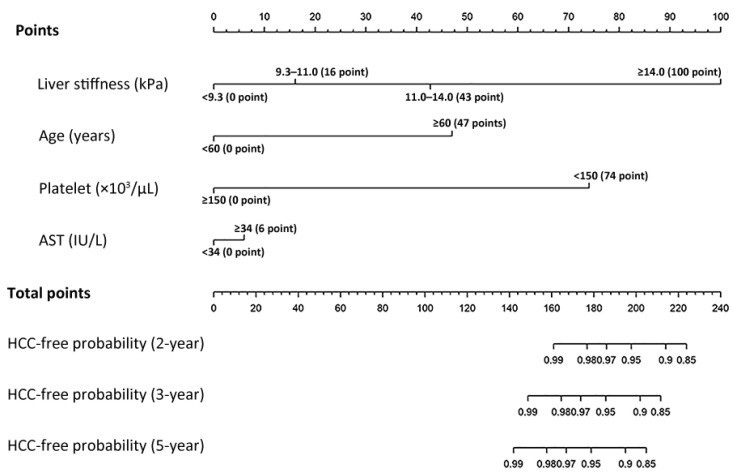
A nomogram of the risk prediction model for HCC development in patients with non-alcoholic fatty liver disease. HCC, hepatocellular carcinoma; AST, aspartate aminotransferase.

Considering the stratified risk of categorized LS, Model 2 was selected as a novel risk prediction model for HCC development among subjects with NAFLD, which categorized LS as <9.3 kPa, 9.3–11.0 kPa, 11.0–14.0 kPa, and ≥14.0 kPa. This model’s score ranged from 0 to 227, and when the sum of the scores was more than 142, 195, 212 and 223 the 5-year probability of developing HCC was calculated to be 1%, 10%, 20% and 30%, respectively (Figure 2 and Table S4).

### 3.5. Internal and External Validation of a New Risk Prediction Model for HCC

Internal validation using bootstrap sampling in the training cohort revealed that the iAUC was also high (0.954) and the 2-, 3- and 5-year AUCs were 0.956, 0.955 and 0.950, respectively (Table 3). Calibration plots for the model for predicting 2-, 3-, and 5-year HCC development showed that the predicted probabilities were very close to the observed incidence rates (Figure S1).

In the validation cohort (*n* = 467), the acceptable performance of the model was verified by analyzing the iAUCs (0.782). The 2-, 3- and 5-year AUC were 0.777, 0.781 and 0.784, respectively (Table 3). The calibration plot for the validation cohort showed that the predicted probabilities were also very close to the observed incidence rates (Figure S1).

The performance and calibration plots of the non-selected models were also acceptable in the internal validation using bootstrap sampling and external validation (Table S5, Figures S2 and S3).

## 4. Discussion

In this retrospective study, we developed and validated a new risk prediction model using potential risk factors for HCC development among patients with NAFLD, by analyzing of multicenter data from the independent high-volume medical centers in the ROK. The two study cohorts included non-significant alcohol-experienced patients with fatty liver, who were evaluated for hepatic fibrosis using TE. Although several recent studies have suggested varying cutoff values [27], we selected the low cutoff of CAP (238 dB/m) to distinguish non-steatosis (S0) according to the previous clinicopathology study [19], due to the possibility of hepatic fat loss in advanced nonalcoholic steatohepatitis and cirrhosis, which have an increased HCC risk. The incidence rate of HCC among subjects in the training and validation cohorts was approximately 1.63 and 8.19 per 1000 patient years, respectively. Multivariate Cox analysis in the training cohort revealed independent risk factors for HCC development such as old age, low platelet counts and LS. After comparing the possible risk prediction models created by combining these risk factors and other variables, a risk prediction model was finally established using age, platelet count, LS and AST. The performance of our newly proposed risk prediction model for NAFLD-HCC was high (iAUC = 0.950) in the internal validation using the bootstrap method and also acceptable (iAUC = 0.728) in the validation cohort.

Our study has several important clinical implications. First, the incidence rate of HCC in our current study was relatively higher than that reported by Kim et al. as 0.23 per 1000 patient years in 8721 patients with NAFLD identified from a health checkup in a healthy population in the ROK [15]. Although the higher incidence rates were reported to range from 1.11 to 45 per 1000 patient years in the subgroups with mainly NASH and cirrhosis [5,12,28,29,30]. Western countries reported incidence rates ranging from 0.08 to 0.6 per 1000 patient years in patients with mainly NAFLD rather than NASH [5,12,28,31], which is relatively lower than the results of our study. Although the exact reason for this phenomenon is unclear, it can be explained in part by the potentially higher proportion of patients at high-risk for NASH with unfavorable medical conditions (such as steatohepatitis and liver fibrosis) in large-volume tertiary medical centers, who require regular follow-up. Indeed, the significant fibrosis burden in our study (28.6% and 36.4%) was higher than those in previous reports (2.8–20.4%) [13,28,32,33]. Although HCC development was not frequently observed, long-term follow-up of a large-group of patients with NAFLD and unfavorable medical conditions could enable the elucidation of risk factors and construction of predictive models.

Second, this study was conducted with a large number of patients who were followed for a long-term period in high-volume hospitals in the ROK. In addition, all subjects in our study had information regarding TE results to assess the degree of liver fibrosis and diagnose NAFLD with higher accuracy and subjectivity [2,16,20,34]. Indeed, to the best of our knowledge, this study is the first to conduct risk stratification of HCC in patients with NAFLD based on TE results. Due to such high accuracy, subjectivity, and reproducibility, various TE-based risk prediction models for hepatitis B-related HCC have been proposed [18,35].

Third, the incidence rate of HCC in the validation cohort was significantly higher than that in the training cohort, probably due to the unfavorable clinical characteristics such as higher prevalence of cirrhosis, higher LS, and higher aminotransferase levels in the validation cohort. Despite of these variable clinical characteristics and follow-up durations between the training and validation cohorts, our newly proposed model showed acceptable performance in the validation cohort. Although the small number of HCC development in the validation cohort (*n* = 9, 1.9%) and relatively short follow-up duration might affect the decreased iAUC in the validation cohort (iAUC = 0.944 to 0.782), the discriminatory performance was still acceptable. In addition, the calibration plot of HCC development indicated that the model provided unbiased estimation results. Moreover, our model showed consistent AUCs at 2, 3 and 5 years in both the training and validation cohorts, suggesting that the good performance of our risk prediction model may be consistent over time.

Fourth, our risk prediction model for HCC development can be easily applied to patients with NAFLD who are on regular medical follow-up, since it uses simple information that is readily available, such as the patient’s age, platelet count and AST level, except LS assessment using TE, which is usually available in tertiary medical centers. The derived risk factors in this study are also well reported in the literature [11,12,13,15,16]. Furthermore, the model uses LS by TE and can indicate the presence of cirrhosis instead. Notably, we revealed that in addition to the high LS values (>11.0 or >14.0 kPa) corresponding to cirrhosis, the LS values corresponding to advanced fibrosis (≥9.6 kPa) are also possible risk factors for the development of HCC in NAFLD. The results suggest that there might be a risk of HCC development in patients with high LS values, even if they do not have cirrhotic features on US. TE results could compensate for the inadequate scan quality of US in patients with obesity or cirrhosis [36].

Our study had some limitations. First, this study was based on a retrospective setting that included only subjects who underwent TE, and could have been potentially subject to selection bias. Second, in addition to the extremely small proportion of subjects with XL probe, the availability of XL probe in only one institutes has limited further analysis regarding XL probe. However, this might limit obese subjects who are in greater need to be examined by TE. Third, we did not consider the on-therapy changes in risk factors, such as fibrosis regression or improvement in necroinflammation after weight reduction. Fourth, it is unclear whether LS by TE represents actual intrahepatic fibrosis, since our data lacked paired liver biopsies. In addition, due to the absence of histological information, we could not investigate the influence of NASH on the risk of HCC in our study. Fifth, the cost of TE can make our risk prediction model difficult for anyone to use clinically. Clinicians may use cirrhosis instead of the highest LS categorization score. Further studies are needed to investigate whether fibrosis assessment based on TE can be substituted with other noninvasive surrogates such as the fibrosis-4 index for application in primary care clinics [2,15].

## 5. Conclusions

This study developed and validated a risk prediction model using simple information that is readily available such as age, platelet count, AST level, and LS by TE, to predict HCC in patients with NAFLD. Despite the heterogeneity of the patient characteristics between the two cohorts, this model showed acceptable performance in the training and validation cohorts. Our risk prediction model may provide information regarding the classification of patients with NAFLD for whom medical follow-up strategies to detect HCC development should be cautioned.

## Figures and Tables

**Table 1:** Baseline characteristics in the training cohort.

Variable	Total (*n* = 2666)	Non-HCC (*n* = 2644, 99.2%)	HCC (*n* = 22, 0.8%)	*p* Value
Age (years)	52.0 (41.0–60.0)	52.0 (41.0–60.0)	70.0 (61.0–72.3)	<0.001
Male sex	1524 (57.2)	1509 (57.1)	15 (68.2)	0.204
Diabetes mellitus	1029 (38.6)	1016 (38.4)	13 (59.1)	0.041
Hypertension	1080 (40.5)	1065 (40.3)	15 (68.2)	0.008
BMI (kg/m^2^)	26.18 (24.17–28.73)	26.2 (24.2–28.7)	27.3 (24.4–29.6)	0.573
Cirrhosis	171 (6.4)	153 (5.8)	18 (81.8)	<0.001
Transient elastography				
LS (kPa)	5.9 (4.6–7.9)	5.9 (4.6–7.8)	21.5 (13.4–33.0)	<0.001
<7.5	1903 (71.4)	1902 (71.9)	1 (4.5)	
7.5–9.3	333 (12.4)	332 (12.6)	1 (4.5)	
9.3–11.0	127 (4.8)	125 (4.7)	2 (9.1)	
11.0–14.0	112 (4.2)	110 (4.2)	2 (9.1)	
≥14.0	191 (7.2)	175 (6.6)	16 (72.8)	
CAP (dB/m)	303 (273–331)	303 (273–331)	290 (256–325)	0.122
Laboratory test results				
Platelet count (×10^3^/μL)	236.0 (200.0–279.0)	237.0 (201.0–279.0)	142.5 (114.8–163.8)	<0.001
AST (IU/L)	33 (24–49)	33 (24–49)	47 (32–62)	0.004
ALT (IU/L)	41 (24–68)	41 (24–69)	32 (20–51)	0.200
Total bilirubin (mg/dL)	0.7 (0.5–0.9)	0.7 (0.5–0.9)	1.0 (0.6–1.5)	0.009
Serum albumin (g/dL)	4.5 (4.3–4.7)	4.5 (4.3–4.7)	4.1 (3.7–4.4)	0.001
Prothrombin time (INR)	0.95 (0.91–1.00)	0.95 (0.91–1.00)	1.08 (1.02–1.19)	0.041
Serum creatinine (mg/dL)	0.78 (0.66–0.92)	0.78 (0.66–0.92)	0.76 (0.69–0.92)	0.837
Gamma-GT (mg/dL)	45 (28–73)	44 (28–73)	75 (53–124)	0.010
ALP (IU/L)	64 (53–79)	64 (52–79)	84 (66–103)	0.001
Triglyceride (mg/dL)	155 (110–208)	155 (110–208)	149 (106–168)	0.499
LDL-cholesterol (mg/dL)	121 (94–148)	121 (94–148)	94 (80–115)	0.002
HDL-cholesterol (mg/dL)	45 (39–52)	45 (39–52)	42 (32–49)	0.131
Total cholesterol (mg/dL)	189 (162–216)	189 (163–216)	150 (134.8–191)	<0.001

Values are expressed as mean ± standard deviation or *n* (%). BMI, body mass index; LS, liver stiffness; CAP, controlled attenuated parameter; AST, aspartate aminotransferase; ALT, alanine aminotransferase; INR, international normalized ratio; gamma-GT; ALP, alkaline phosphatase; gamma-glutamyl transferase; LDL, low-density lipoprotein; HDL, high-density lipoprotein.

**Table 2:** Cox multivariate analysis for HCC development.

Variable	Univariate *p* Value	Multivariate Analysis	
		*p* Value	Hazard Ratio (95% CI)
Age ≥ 60 (vs. <60) years	<0.001	0.004	9.143 (2.055, 40.684)
Liver stiffness ≥ 9.3 (vs. <9.3) kPa	<0.001	0.001	13.757 (2.826, 66.955)
Platelet count < 150 (vs. ≥150) × 10^3^/μL	<0.001	0.014	3.670 (1.295, 10.402)
AST ≥ 34 (vs. <34) IU/L	0.068	0.395	1.583 (0.549, 4.560)
Serum albumin < 3.4 (vs. ≥3.4) g/dL	<0.001	0.161	2.699 (0.674, 10.803)
Total bilirubin ≥ 2.0 (vs. <2.0) mg/dL	<0.001	0.566	1.485 (0.385, 5.737)
Total cholesterol ≥ 168 (vs. <168) mg/dL	<0.001	0.259	0.560 (0.204, 1.534)

CI, confidence interval; AST, aspartate aminotransferase.

**Table 3:** Time-dependent AUC of the selected risk prediction model in the training cohort, internal validation using the bootstrap method, and external validation.

AUC	Training Cohort	Internal Validation (Bootstrap)	Validation Cohort
iAUC	0.944 (0.909, 0.979)	0.954 (0.916, 0.982)	0.782 (0.610, 0.954)
2 yr AUC	0.948 (0.917, 0.979)	0.956 (0.922, 0.982)	0.777 (0.606, 0.948)
3 yr AUC	0.947 (0.914, 0.980)	0.955 (0.920, 0.983)	0.781 (0.614, 0.948)
5 yr AUC	0.939 (0.900, 0.978)	0.950 (0.909, 0.983)	0.784 (0.619, 0.949)

Values are expressed as AUC (95% confidence intervals). AUC, area under the receiver operating characteristic curve; iAUC, integrated AUC.

## Data Availability

The data presented in this study are available on request from the corresponding author. The data are not publicly available due to patients’ privacy issue.

## Supplementary Materials

The following are available online at https://www.mdpi.com/article/10.3390/cancers13184567/s1, Table S1: Comparison of baseline characteristics between the training and validation cohorts, Table S2: Univariate Cox regression for the development of hepatocellular carcinoma in the training cohort, Table S3: Cox multivariate analysis for the development of hepatocellular carcinoma in patients with non-alcoholic fatty liver disease using variables incorporated in the prediction model, Table S4: Nomogram for each risk prediction model for HCC development, Table S5: Time-dependent AUCs and iAUC of each risk prediction model in the training cohort, internal validation using the bootstrap method, and external validation, Figure S1: Calibration plots of the risk prediction model at 2-, 3-, and 5-year; iAUC and time-dependent receiver operational characteristics curves in training (A) and validation cohort (B). AUC, area under the receiver operational characteristics curves; iAUC, integrated AUC, Figure S2: Calibration plots, iAUCs and time-dependent receiver operational characteristics curves of prediction model 1 (A) and 3 (B) in the training cohort, Figure S3: Calibration plots, iAUCs and time-dependent receiver operational characteristics curves of prediction model 1 (A) and 3 (B) in the validation cohort.

## References

[1] Younossi Z.M., Koenig A.B., Abdelatif D., Fazel Y., Henry L., Wymer M. (2016). Global epidemiology of nonalcoholic fatty liver disease-meta-analytic assessment of prevalence, incidence, and outcomes. Hepatology.

[2] Kang S.H., Lee H.W., Yoo J.J., Cho Y., Kim S.U., Lee T.H., Jang B.K., Kim S.G., Ahn S.B., Kim H. (2021). Kasl clinical practice guidelines: Management of nonalcoholic fatty liver disease. Clin. Mol. Hepatol..

[3] Park S.H., Plank L.D., Suk K.T., Park Y.E., Lee J., Choi J.H., Heo N.Y., Park J., Kim T.O., Moon Y.S. (2020). Trends in the prevalence of chronic liver disease in the korean adult population, 1998–2017. Clin. Mol. Hepatol..

[4] Younossi Z., Stepanova M., Ong J.P., Jacobson I.M., Bugianesi E., Duseja A., Eguchi Y., Wong V.W., Negro F., Yilmaz Y. (2019). Nonalcoholic steatohepatitis is the fastest growing cause of hepatocellular carcinoma in liver transplant candidates. Clin. Gastroenterol. Hepatol..

[5] Huang D.Q., El-Serag H.B., Loomba R. (2021). Global epidemiology of nafld-related hcc: Trends, predictions, risk factors and prevention. Nat. Rev. Gastroenterol. Hepatol..

[6] Yang J.D., Hainaut P., Gores G.J., Amadou A., Plymoth A., Roberts L.R. (2019). A global view of hepatocellular carcinoma: Trends, risk, prevention and management. Nat. Rev. Gastroenterol. Hepatol..

[7] Choi J., Han S., Kim N., Lim Y.S. (2017). Increasing burden of liver cancer despite extensive use of antiviral agents in a hepatitis b virus-endemic population. Hepatology.

[8] Kim Y.A., Lee Y.-R., Park J., Oh I.-H., Kim H., Yoon S.-J., Park K. (2020). Socioeconomic burden of cancer in korea from 2011 to 2015. Cancer Res. Treat..

[9] Wong R.J., Cheung R., Ahmed A. (2014). Nonalcoholic steatohepatitis is the most rapidly growing indication for liver transplantation in patients with hepatocellular carcinoma in the U.S. Hepatology.

[10] Cho E.J., Kwack M.S., Jang E.S., You S.J., Lee J.H., Kim Y.J., Yoon J.H., Lee H.S. (2011). Relative etiological role of prior hepatitis b virus infection and nonalcoholic fatty liver disease in the development of non-b non-c hepatocellular carcinoma in a hepatitis b-endemic area. Digestion.

[11] Ascha M.S., Hanouneh I.A., Lopez R., Tamimi T.A., Feldstein A.F., Zein N.N. (2010). The incidence and risk factors of hepatocellular carcinoma in patients with nonalcoholic steatohepatitis. Hepatology.

[12] Kanwal F., Kramer J.R., Mapakshi S., Natarajan Y., Chayanupatkul M., Richardson P.A., Li L., Desiderio R., Thrift A.P., Asch S.M. (2018). Risk of hepatocellular cancer in patients with non-alcoholic fatty liver disease. Gastroenterology.

[13] Kawamura Y., Arase Y., Ikeda K., Seko Y., Imai N., Hosaka T., Kobayashi M., Saitoh S., Sezaki H., Akuta N. (2012). Large-scale long-term follow-up study of japanese patients with non-alcoholic fatty liver disease for the onset of hepatocellular carcinoma. Am. J. Gastroenterol..

[14] Oh H., Jun D.W., Saeed W.K., Nguyen M.H. (2016). Non-alcoholic fatty liver diseases: Update on the challenge of diagnosis and treatment. Clin. Mol. Hepatol..

[15] Kim G.A., Lee H.C., Choe J., Kim M.J., Lee M.J., Chang H.S., Bae I.Y., Kim H.K., An J., Shim J.H. (2018). Association between non-alcoholic fatty liver disease and cancer incidence rate. J. Hepatol..

[16] Lee H.W., Park S.Y., Kim S.U., Jang J.Y., Park H., Kim J.K., Lee C.K., Chon Y.E., Han K.H. (2016). Discrimination of nonalcoholic steatohepatitis using transient elastography in patients with nonalcoholic fatty liver disease. PLoS ONE.

[17] Zhang X., Wong G.L., Wong V.W. (2020). Application of transient elastography in nonalcoholic fatty liver disease. Clin. Mol. Hepatol..

[18] Lee H.W., Yoo E.J., Kim B.K., Kim S.U., Park J.Y., Kim D.Y., Ahn S.H., Han K.H. (2014). Prediction of development of liver-related events by transient elastography in hepatitis b patients with complete virological response on antiviral therapy. Am. J. Gastroenterol..

[19] Sasso M., Beaugrand M., de Ledinghen V., Douvin C., Marcellin P., Poupon R., Sandrin L., Miette V. (2010). Controlled attenuation parameter (cap): A novel vcte™ guided ultrasonic attenuation measurement for the evaluation of hepatic steatosis: Preliminary study and validation in a cohort of patients with chronic liver disease from various causes. Ultrasound Med. Biol..

[20] Chon Y.E., Jung K.S., Kim S.U., Park J.Y., Park Y.N., Kim D.Y., Ahn S.H., Chon C.Y., Lee H.W., Park Y. (2014). Controlled attenuation parameter (cap) for detection of hepatic steatosis in patients with chronic liver diseases: A prospective study of a native korean population. Liver Int..

[21] Hu Y.Y., Dong N.L., Qu Q., Zhao X.F., Yang H.J. (2018). The correlation between controlled attenuation parameter and metabolic syndrome and its components in middle-aged and elderly nonalcoholic fatty liver disease patients. Medicine.

[22] Lee H.W., Chon Y.E., Kim S.U., Kim B.K., Park J.Y., Kim D.Y., Ahn S.H., Jung K.S., Park Y.N., Han K.H. (2016). Predicting liver-related events using transient elastography in chronic hepatitis c patients with sustained virological response. Gut Liver.

[23] Jung K.S., Kim S.U. (2012). Clinical applications of transient elastography. Clin. Mol. Hepatol..

[24] Lee Y.H., Jung K.S., Kim S.U., Yoon H.J., Yun Y.J., Lee B.W., Kang E.S., Han K.H., Lee H.C., Cha B.S. (2015). Sarcopaenia is associated with nafld independently of obesity and insulin resistance: Nationwide surveys (knhanes 2008–2011). J. Hepatol..

[25] Iasonos A., Schrag D., Raj G.V., Panageas K.S. (2008). How to build and interpret a nomogram for cancer prognosis. J. Clin. Oncol..

[26] Heagerty P.J., Zheng Y. (2005). Survival model predictive accuracy and roc curves. Biometrics.

[27] Karlas T., Petroff D., Sasso M., Fan J.G., Mi Y.Q., de Lédinghen V., Kumar M., Lupsor-Platon M., Han K.H., Cardoso A.C. (2017). Individual patient data meta-analysis of controlled attenuation parameter (cap) technology for assessing steatosis. J. Hepatol..

[28] Alexander M., Loomis A.K., van der Lei J., Duarte-Salles T., Prieto-Alhambra D., Ansell D., Pasqua A., Lapi F., Rijnbeek P., Mosseveld M. (2019). Risks and clinical predictors of cirrhosis and hepatocellular carcinoma diagnoses in adults with diagnosed nafld: Real-world study of 18 million patients in four european cohorts. BMC Med..

[29] Ioannou G.N., Green P., Lowy E., Mun E.J., Berry K. (2018). Differences in hepatocellular carcinoma risk, predictors and trends over time according to etiology of cirrhosis. PLoS ONE.

[30] Hsiang J.C., Bai W.W., Raos Z., Stableforth W., Upton A., Selvaratnam S., Gane E.J., Gerred S.J. (2015). Epidemiology, disease burden and outcomes of cirrhosis in a large secondary care hospital in south auckland, new zealand. Intern. Med. J..

[31] Adams L.A., Lymp J.F., St Sauver J., Sanderson S.O., Lindor K.D., Feldstein A., Angulo P. (2005). The natural history of nonalcoholic fatty liver disease: A population-based cohort study. Gastroenterology.

[32] Seko Y., Sumida Y., Tanaka S., Mori K., Taketani H., Ishiba H., Hara T., Okajima A., Umemura A., Nishikawa T. (2017). Development of hepatocellular carcinoma in japanese patients with biopsy-proven non-alcoholic fatty liver disease: Association between pnpla3 genotype and hepatocarcinogenesis/fibrosis progression. Hepatol. Res..

[33] Ito T., Ishigami M., Ishizu Y., Kuzuya T., Honda T., Hayashi K., Nishimura D., Toyoda H., Kumada T., Goto H. (2019). Utility and limitations of noninvasive fibrosis markers for predicting prognosis in biopsy-proven japanese non-alcoholic fatty liver disease patients. J. Gastroenterol. Hepatol..

[34] Lee H.W., Kim B.K., Kim S.U., Park J.Y., Kim D.Y., Ahn S.H., Kim K.J., Han K.H. (2017). Prevalence and predictors of significant fibrosis among subjects with transient elastography-defined nonalcoholic fatty liver disease. Dig. Dis. Sci..

[35] Jung K.S., Kim S.U., Song K., Park J.Y., Kim D.Y., Ahn S.H., Kim B.K., Han K.H. (2015). Validation of hepatitis b virus-related hepatocellular carcinoma prediction models in the era of antiviral therapy. Hepatology.

[36] Loomba R., Lim J.K., Patton H., El-Serag H.B. (2020). Aga clinical practice update on screening and surveillance for hepatocellular carcinoma in patients with nonalcoholic fatty liver disease: Expert review. Gastroenterology.

