# Supplementary material for: Liver Stiffness-Based Risk Prediction Model for Hepatocellular Carcinoma in Patients with Nonalcoholic Fatty Liver Disease
Source: Cancers (Basel). 2021 Sep 11;13(18):4567. doi: 10.3390/cancers13184567 (PMC8472221; doi:10.3390/cancers13184567)
Supplement: Supplementary file 1 [file cancers-13-04567-s001.zip › cancers-1364685-supplementary.pdf]

cancers MDPI

Supplementary Materials: Liver Stiffness-Based Risk Prediction Model for Hepatocellular Carcinoma in Patients with Nonalcoholic Fatty Liver Disease

Jae Seung Lee, Dong Hyun Sinn, Soo Young Park, Hye Jung Shin, Hye Won Lee, Beom Kyung Kim, Jun Yong Park, Do Young Kim, Sang Hoon Ahn, Joo Hyun Oh, Jung Il Lee and Seung Up Kim

Table S1. Comparison of baseline characteristics between the training and validation cohorts.

Variable	Training cohort (n=2,666)	Validation cohort (n=467)	*P* value
Age (years)	52.0 (41.0–60.0)	53.0 (38.0–62.0)	0.222
Male sex	1,524 (57.2)	244 (52.3)	0.048
Diabetes mellitus	1,029 (38.6)	100 (21.4)	<0.001
Hypertension	1,080 (40.5)	100 (21.4)	<0.001
Body mass index (kg/m^2)	26.18 (24.17–28.73)	27.74 (25.80–29.40)	<0.001
Liver cirrhosis*	171 (6.4)	44 (9.4)	0.018
Transient elastography			
LS (kPa)	5.9 (4.6–7.9)	6.5 (5.0–9.6)	0.006
< 7.5	1,903 (71.4)	297 (63.6)	
7.5–9.3	333 (12.4)	45 (9.6)	
9.3–11.0	127 (4.8)	44 (9.4)	<0.001
11.0–14.0	112 (4.2)	24 (5.2)	
≥ 14.0	191 (7.2)	57 (12.2)	
CAP (dB/m)	303 (273–331)	300.0 (275–325)	0.207
Laboratory test results			
Platelet count (×10^3/μL)	236.0 (200.0–279.0)	240.5 (198.0–285.5)	0.097
AST (IU/L)	33 (24–49)	47 (31–74)	<0.001
ALT (IU/L)	41 (24–68)	57 (30–98)	<0.001
Total bilirubin (mg/dL)	0.7 (0.5–0.9)	0.61 (0.44–0.85)	<0.001
Serum albumin (g/dL)	4.5 (4.3–4.7)	4.7 (4.4–4.9)	<0.001
Prothrombin time (INR)	0.95 (0.91–1.00)	1.00 (0.97–1.05)	<0.001
Serum creatinine (mg/dL)	0.78 (0.66–0.92)	0.77 (0.65–0.91)	0.012
Gamma-GT (mg/dL)	45 (28 -73)	49 (44–59)	<0.001
Alkaline Phosphatase (IU/L)	64 (53 -79)	75 (62–91)	<0.001
Triglyceride (mg/dL)	155 (110–208)	154 (107–208)	0.471
LDL-cholesterol (mg/dL)	121 (94–148)	116 (87–147)	0.002
HDL-cholesterol (mg/dL)	45 (39–52)	46 (39–55)	0.039
Total cholesterol (mg/dL)	189 (162–216)	180 (149–213)	0.000

Values are expressed as median (interquartile range) or n (%). LS, liver stiffness; CAP, controlled attenuated parameter; AST, aspartate aminotransferase; ALT, alanine aminotransferase; INR, international normalized ratio; gamma-GT, gamma-glutamyl transferase; LDL, low-density lipoprotein; HDL, high-density lipoprotein; HCC, hepatocellular carcinoma.

Table S2. Univariate Cox regression for the development of hepatocellular carcinoma in the training cohort.

Variable	*P* value	Hazard ratio (95% CI)	Variable	*P* value	Hazard ratio (95% CI)
Age (years)	<0.001	1.119 (1.076, 1.163)	Platelet count (×10³/μL)	<0.001	0.974 (0.967, 0.982)
≥55 (vs. <55)	0.001	12.750 (2.980, 54.555)	<150 (vs. ≥150)	<0.001	7.875 (3.205, 19.345)
≥60 (vs. <60)	<0.001	11.223 (3.798, 33.165)	<166 (vs. ≥166)	<0.001	31.759 (10.616, 95.010)
≥65 (vs. <65)	<0.001	9.347 (3.919, 22.292)	Aspartate aminotransferase (IU/L)	0.002	1.013 (1.005, 1.022)
≥70 (vs. <70)	<0.001	14.595 (6.283, 33.907)	≥34 (vs. <34)	0.068	2.310 (0.942, 5.665)
Male sex	0.254	1.686 (0.687, 4.135)	≥38 (vs. <38)	0.012	3.181 (1.297, 7.804)
Diabetes mellitus	0.157	1.850 (0.789, 4.341)	Alanine aminotransferase (IU/L)	0.267	0.992 (0.978, 1.006)
Hypertension	0.054	2.423 (0.984, 5.963)	≥34 (vs. <34)	0.439	0.719 (0.312, 1.659)
Body mass index (kg/m²)	0.610	1.029 (0.921, 1.150)	Total bilirubin (mg/dL)	<0.001	4.786 (2.491, 9.197)
≥18 (vs. <18)	0.214	0.161 (0.009, 2.866)	≥2.0 (vs. <2.0)	<0.001	10.939 (3.231, 37.033)
≥25 (vs. <25)	0.897	1.061 (0.433, 2.605)	≥0.9 (vs. <0.9)	0.001	4.496 (1.886, 10.719)
Cirrhosis*	<0.001	65.851 (22.268, 194.732)	Albumin (g/dL)	<0.001	0.093 (0.043, 0.203)
Liver stiffness measurement (kPa)	<0.001	1.089 (1.071, 1.108)	<3.4 (vs. ≥3.4)	<0.001	21.432 (6.250, 73.486)
≥8.3 (vs. <8.3)	<0.001	34.09 (7.968, 145.856)	<4.5 (vs. ≥4.5)	0.002	9.901 (2.314, 42.365)
≥9.3 (vs. <9.3)	<0.001	51.475 (12.030, 220.256)	Prothrombin time (INR)	0.088	2.752 (0.860, 8.803)
≥11.0 (vs. <11)	<0.001	35.141 (11.891, 103.854)	≥1.5 (vs. <1.5)	0.610	2.152 (0.113, 40.829)
≥14.0 (vs. <14)	<0.001	34.212 (13.384, 87.453)	Serum creatinine (mg/dL)	0.896	0.888 (0.150, 5.268)
Liver stiffness group (vs. <8.3 kPa)	-	ref	Gamma-glutamyl transferase (mg/dL)	0.008	1.004 (1.001, 1.006)
≥8.3, <11.0	0.051	7.072 (0.996, 50.209)	Alkaline phosphatase (IU/L)	0.001	1.007 (1.003, 1.011)
≥11.0, <14.0	0.004	18.623 (2.623, 132.224)	≥150 (vs. <150)	0.127	4.771 (0.640, 35.573)
≥14.0	<0.001	88.322 (20.306, 384.157)	Triglyceride (mg/dL)	0.558	0.998 (0.992, 1.004)
Liver stiffness group (vs. <9.3 kPa)	-	ref	LDL-cholesterol (mg/dL)	0.061	0.98 (0.960, 1.001)
≥9.3, <11.0	0.005	17.004 (2.395, 120.744)	HDL-cholesterol (mg/dL)	0.148	0.954 (0.896, 1.017)
≥11.0, <14.0	0.003	20.313 (2.861, 144.234)	Total cholesterol (mg/dL)	0.001	0.978 (0.966, 0.990)
≥14.0	<0.001	92.645 (21.300, 402.958)	≥168 (vs. <168)	0.001	4.696 (1.914, 11.518)
Controlled attenuated parameter (dB/m)	0.114	0.990 (0.979, 1.002)			

Additional Cox regression analyses were performed on continuous variables after changing them to dichotomous categorical variables. * Cirrhosis are diagnosed by imaging studies such as abdomen ultrasonography or computed tomography.INR, international normalized ratio; LDL, low-density lipoprotein; HDL, high-density lipoprotein.

Table S3. Cox multivariate analysis for the development of hepatocellular carcinoma in patients with non-alcoholic fatty liver disease using variables incorporated in the prediction model.

Variable		Model 1		Model 2		Model 3	
		HR (95% CI)	*p* Value	HR (95% CI)	*p* Value	HR (95% CI)	*p* Value
Age (year)	≥60	10.377 (2.344, 45.935)	0.002	10.566 (2.381, 46.896)	0.002	10.056 (2.273, 44.494)	0.002
Platelet count (×10³/μL)	<150	4.300 (1.557, 11.876)	0.005	3.246 (1.114, 9.459)	0.031	4.985 (1.835, 13.543)	0.002
AST (IU/L)	≥34	1.691 (0.585, 4.889)	0.332	1.565 (0.539, 4.547)	0.410	1.589 (0.560, 4.512)	0.385
Liver Stiffness† (kPa)	≥11.0	14.102 (3.615, 55.016)	0.000				
	<9.3			Reference	-		
	9.3–11.0			4.377 (0.381, 50.239)	0.236		
	11.0–14.0			10.617 (1.42, 79.399)	0.021		
	≥14.0			25.802 (4.928, 135.093)	0.000		
	<7.5					Reference	-
	7.5–9.6					6.989 (0.623, 78.459)	0.115
	≥ 9.6					23.026 (2.776, 190.992)	0.004

HR, hazard ratio; CI, confidence interval; AST, aspartate aminotransferase. † Measured by transient elastography (FibroScan®, EchoSens, Paris, France).

Table S4. Nomogram for each risk prediction model for HCC development.

Models	Model 1 (max 263 points)		Model 2 (max 227 points)		Model 3 (max 240 Points)	
Covariates	Category	Points *	Category	Points *	Category	Points *
Liver stiffness † (kPa)	≥11.0	100	9.3–11.0	16	7.5–9.6	62
			11.0–14.0	43	≥9.6	100
			≥14.0	100		
Age (years)	≥60	88	≥60	47	≥60	74
Platelet counts (×10³/μL)	<150	55	<150	74	<150	51
AST (IU/L)	≥34	20	≥34	6	≥34	15
Time point	Total Points	HCC-free probability	Total Points	HCC-free probability	Total Points	HCC-free probability
At 2 years	168	0.99	161	0.99	164	0.99
	257	0.90	214	0.90	239	0.90
At 3 years	148	0.99	149	0.99	148	0.99
	237	0.90	202	0.90	223	0.90
	254	0.85	212	0.85	236	0.85
	263	0.81	219	0.80	240	0.83
			225	0.75		
			227	0.73		
At 5 years	138	0.99	142	0.99	139	0.99
	199	0.95	179	0.95	191	0.95
	227	0.90	195	0.90	214	0.90
	243	0.85	205	0.85	228	0.85
	255	0.80	212	0.80	238	0.80
	263	0.76	218	0.75	240	0.79
			223	0.70		
			227	0.65		

HCC, hepatocellular carcinoma; AST, aspartate aminotransferase. *Zero point, if the condition is not met. †Measured by transient elastography (FibroScan®, EchoSens, Paris, France).

Table S5. Time-dependent AUCs and iAUC of each risk prediction model in the training cohort, internal validation using the bootstrap method, and external validation.

**Model 1**	**Training cohort**	**Internal validation (bootstrap)**	**Validation cohort**
iAUC	0.939 (0.902, 0.976)	0.945 (0.906, 0.979)	0.762 (0.582, 0.942)
2yr AUC	0.943 (0.908, 0.978)	0.949 (0.911, 0.978)	0.755 (0.579, 0.931)
3yr AUC	0.941 (0.904, 0.978)	0.947 (0.909, 0.978)	0.758 (0.584, 0.932)
5yr AUC	0.933 (0.892, 0.974)	0.941 (0.898, 0.978)	0.787 (0.609, 0.965)
Model 2	Training cohort	Internal validation (bootstrap)	Validation cohort
iAUC	0.944 (0.909, 0.979)	0.954 (0.916, 0.982)	0.782 (0.610, 0.954)
2yr AUC	0.948 (0.917, 0.979)	0.956 (0.922, 0.982)	0.777 (0.606, 0.948)
3yr AUC	0.947 (0.914, 0.980)	0.955 (0.920, 0.983)	0.781 (0.614, 0.948)
5yr AUC	0.939 (0.900, 0.978)	0.950 (0.909, 0.983)	0.784 (0.619, 0.949)
Model 3	Training cohort	Internal validation (bootstrap)	Validation cohort
iAUC	0.942 (0.907, 0.977)	0.950 (0.909, 0.977)	0.729 (0.531, 0.927)
2yr AUC	0.945 (0.912, 0.978)	0.951 (0.913, 0.977)	0.722 (0.528, 0.916)
3yr AUC	0.944 (0.909, 0.979)	0.950 (0.912, 0.977)	0.725 (0.531, 0.919)
5yr AUC	0.940 (0.903, 0.977)	0.947 (0.902, 0.976)	0.728 (0.550, 0.906)

Values are expressed as AUC (95% confidence interval). AUC, area under the receiver operating characteristic curve; iAUC, integrated AUC.

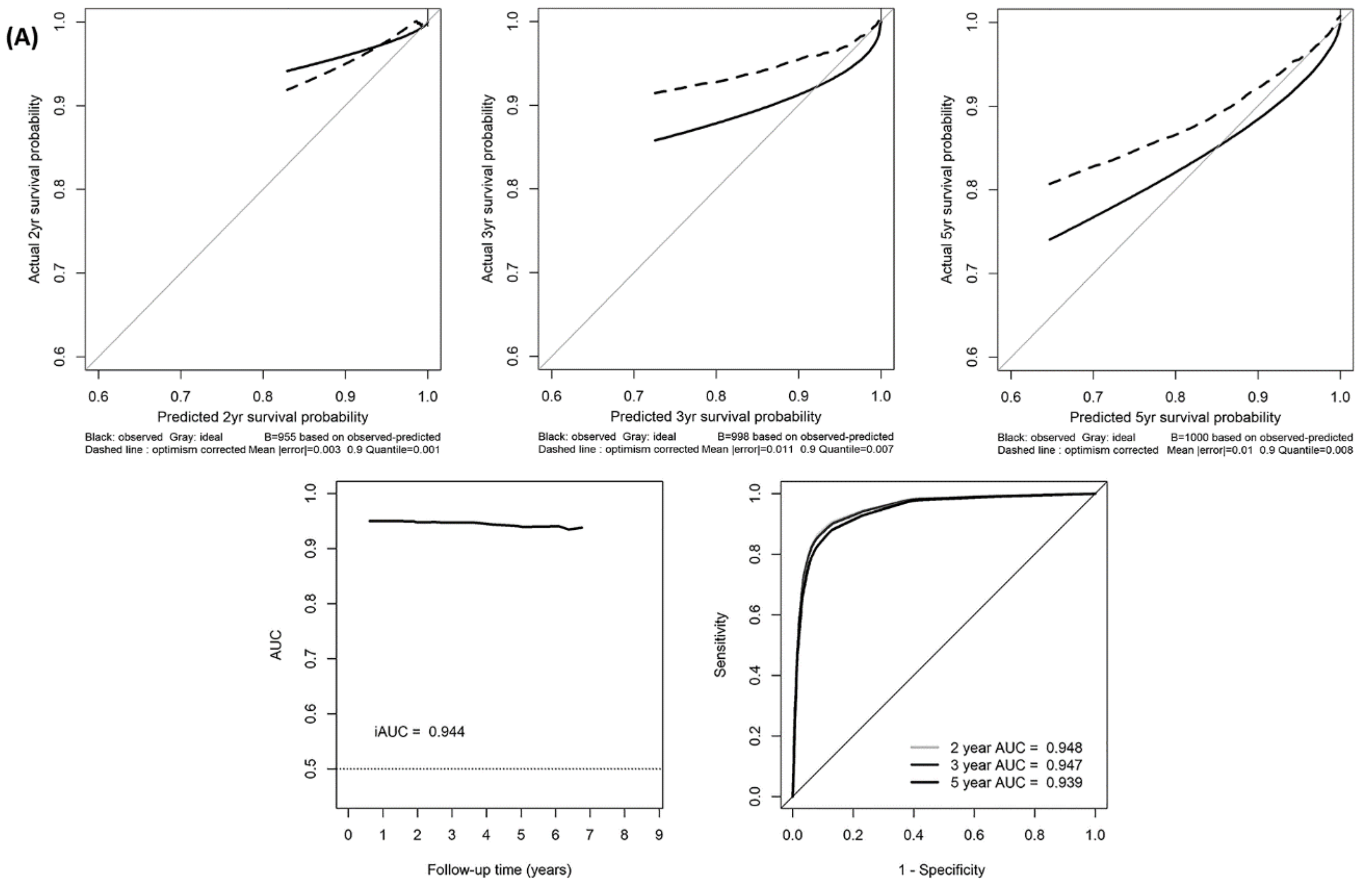

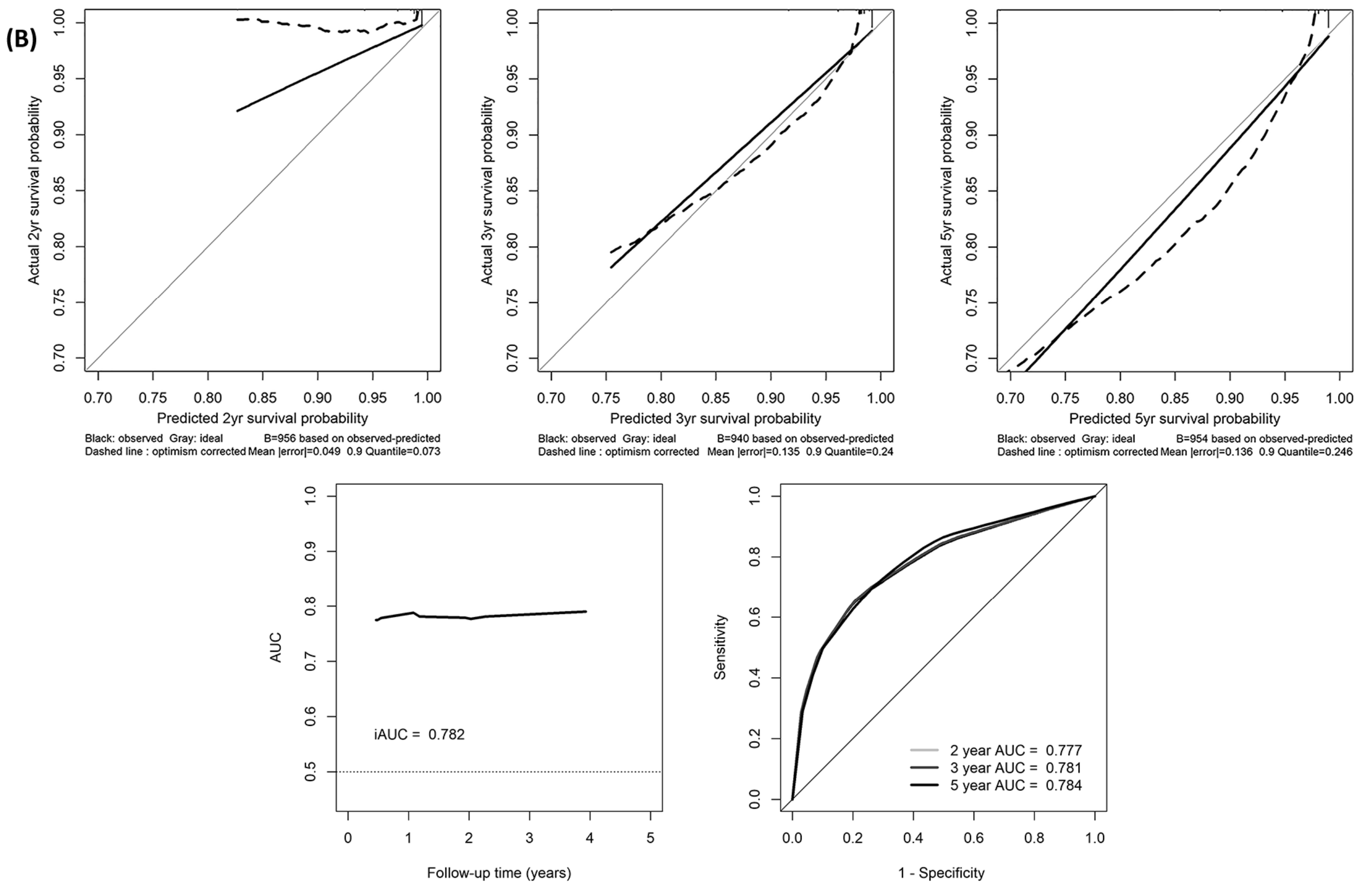

Figure S1. Calibration plots of the risk prediction model at 2-, 3-, and 5-year; iAUC and time-dependent receiver operational characteristics curves in training (**A**) and validation cohort (**B**). AUC, area under the receiver operational char-acteristics curves; iAUC, integrated AUC.

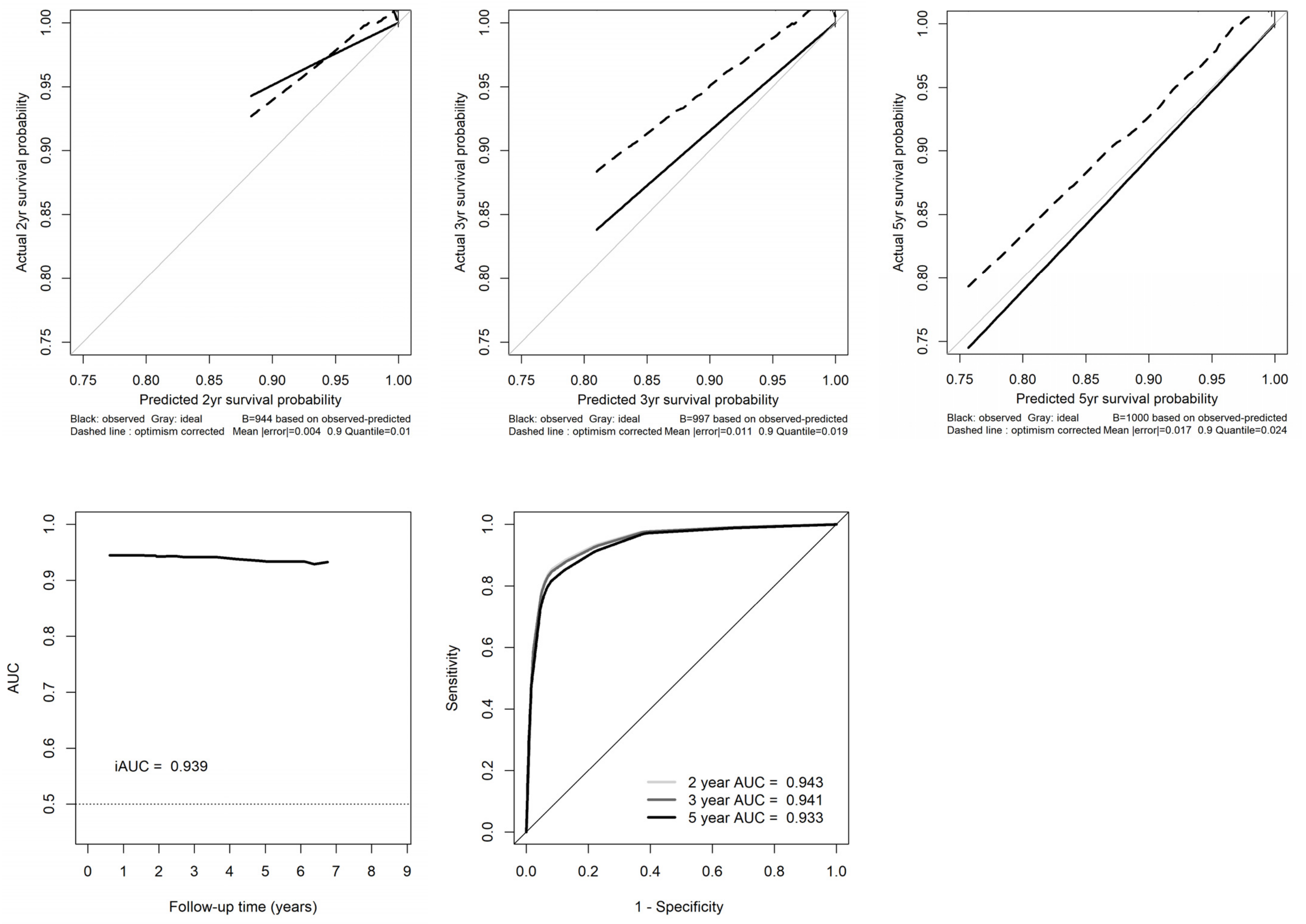

Actual 2yr survival probability
Predicted 2yr survival probability
Black: observed Gray: ideal B=944 based on observed-predicted
Dashed line : optimism corrected Mean |error|=0.004 0.9 Quantile=0.01
Actual 3yr survival probability
Predicted 3yr survival probability
Black: observed Gray: ideal B=997 based on observed-predicted
Dashed line : optimism corrected Mean |error|=0.011 0.9 Quantile=0.019
Actual 5yr survival probability
Predicted 5yr survival probability
Black: observed Gray: ideal B=1000 based on observed-predicted
Dashed line : optimism corrected Mean |error|=0.017 0.9 Quantile=0.024
AUC
iAUC = 0.939
Follow-up time (years)
Sensitivity
1 - Specificity
2 year AUC = 0.943
3 year AUC = 0.941
5 year AUC = 0.933

(**A**)

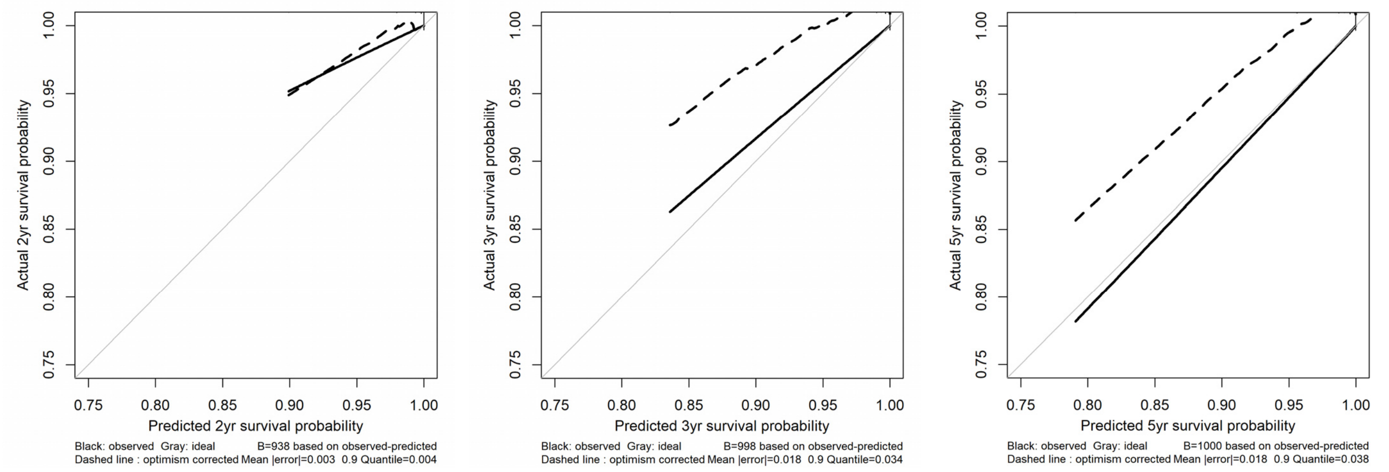

Actual 2yr survival probability
Predicted 2yr survival probability
Black: observed Gray: ideal B=938 based on observed-predicted
Dashed line : optimism corrected Mean |error|=0.003 0.9 Quantile=0.004
Actual 3yr survival probability
Predicted 3yr survival probability
Black: observed Gray: ideal B=998 based on observed-predicted
Dashed line : optimism corrected Mean |error|=0.018 0.9 Quantile=0.034
Actual 5yr survival probability
Predicted 5yr survival probability
Black: observed Gray: ideal B=1000 based on observed-predicted
Dashed line : optimism corrected Mean |error|=0.018 0.9 Quantile=0.038

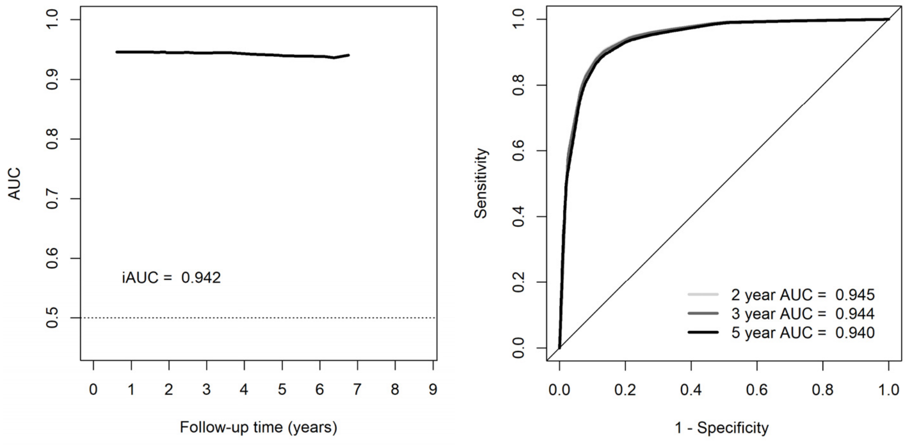

(**B**)

Figure S2. Calibration plots, iAUCs and time-dependent receiver operational characteristics curves of prediction model 1 (**A**) and 3 (**B**) in the training cohort. AUC, area under the receiver operational curve; iAUC, integrated AUC.

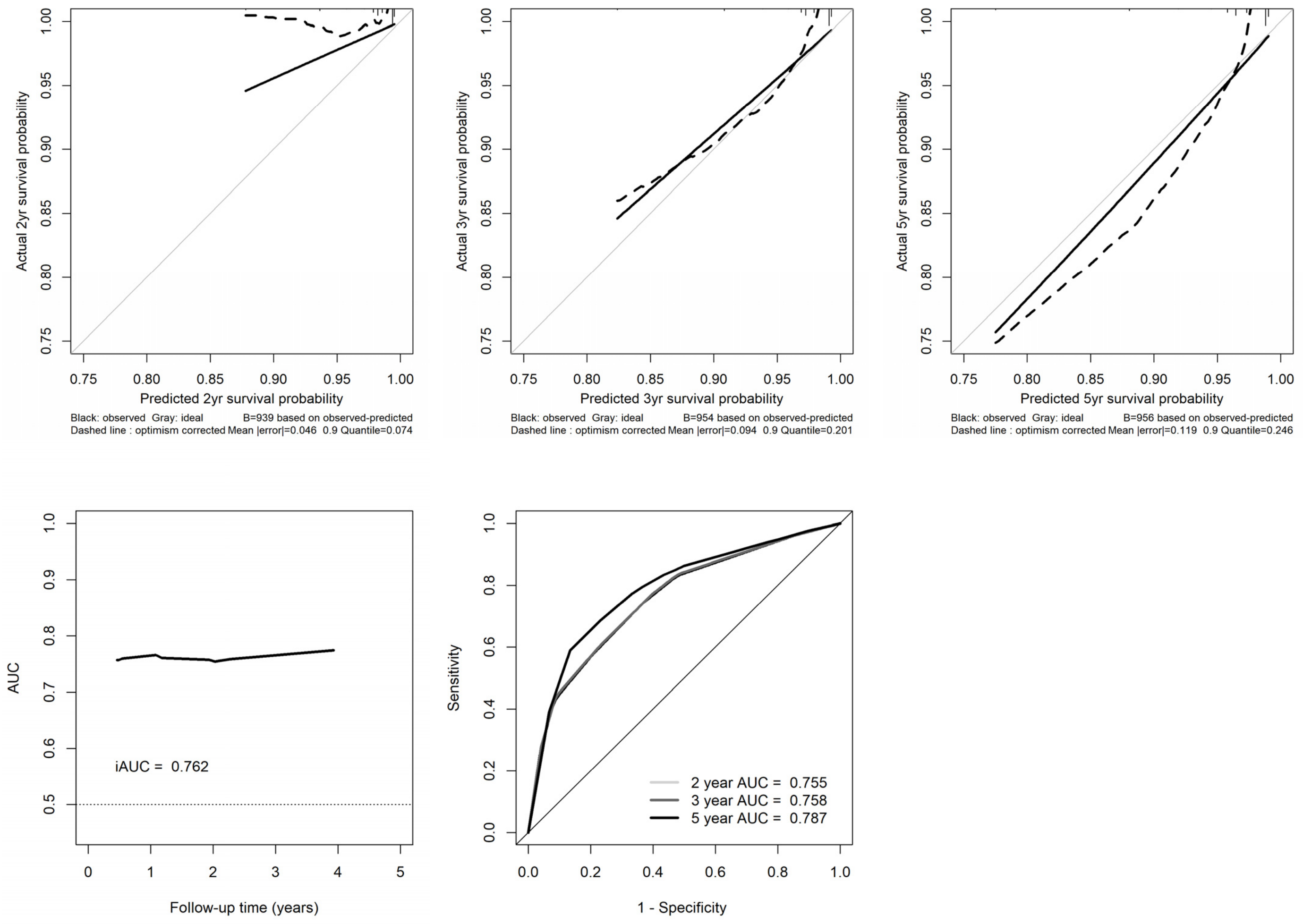

(**A**)

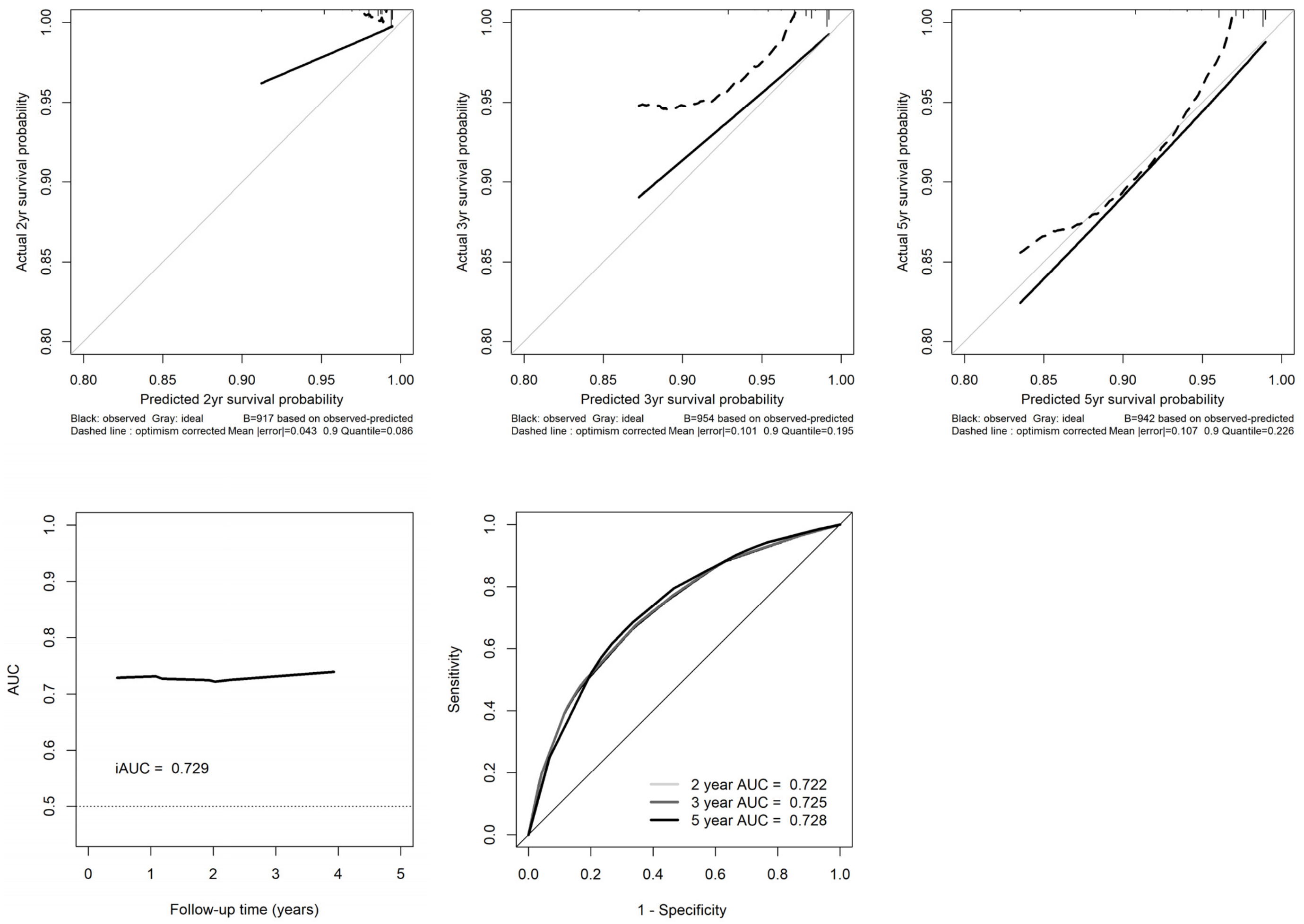

(**B**)

Figure S3. Calibration plots, iAUCs and time-dependent receiver operational characteristics curves of prediction model 1 (**A**) and 3 (**B**) in the validation cohort. AUC, area under the receiver operational curve; iAUC, integrated AUC.
